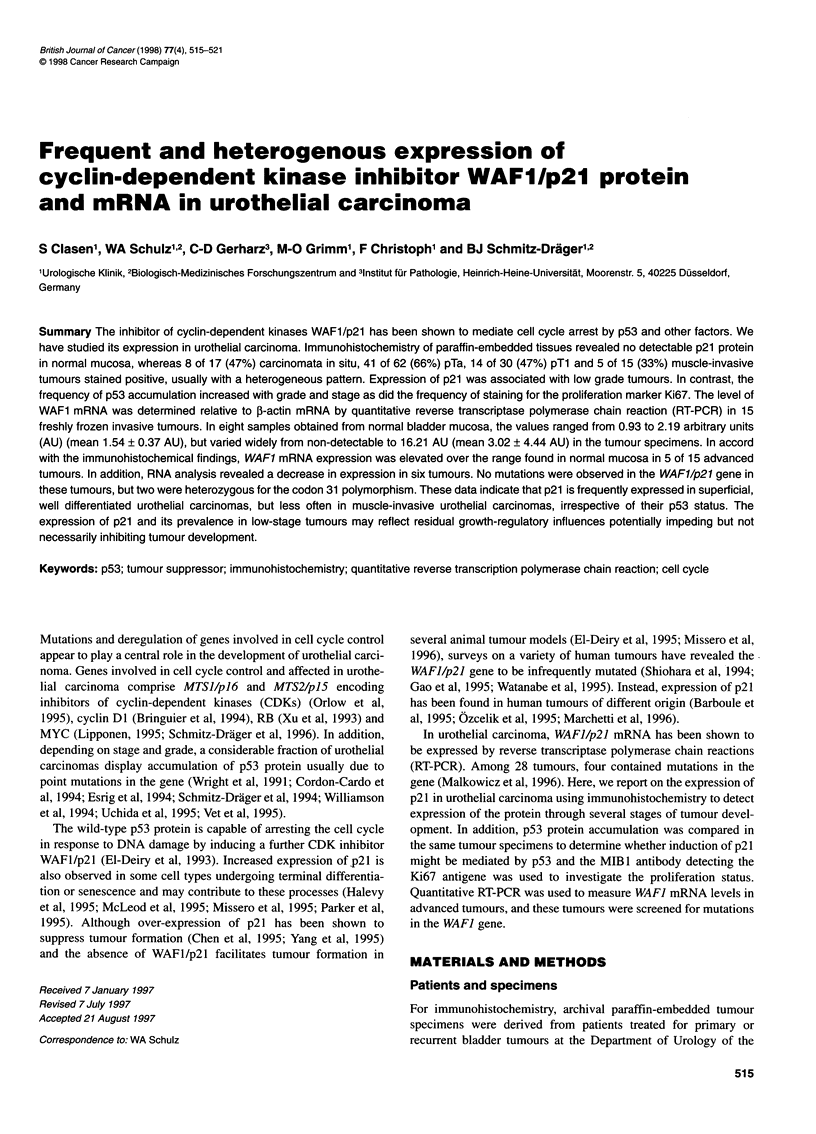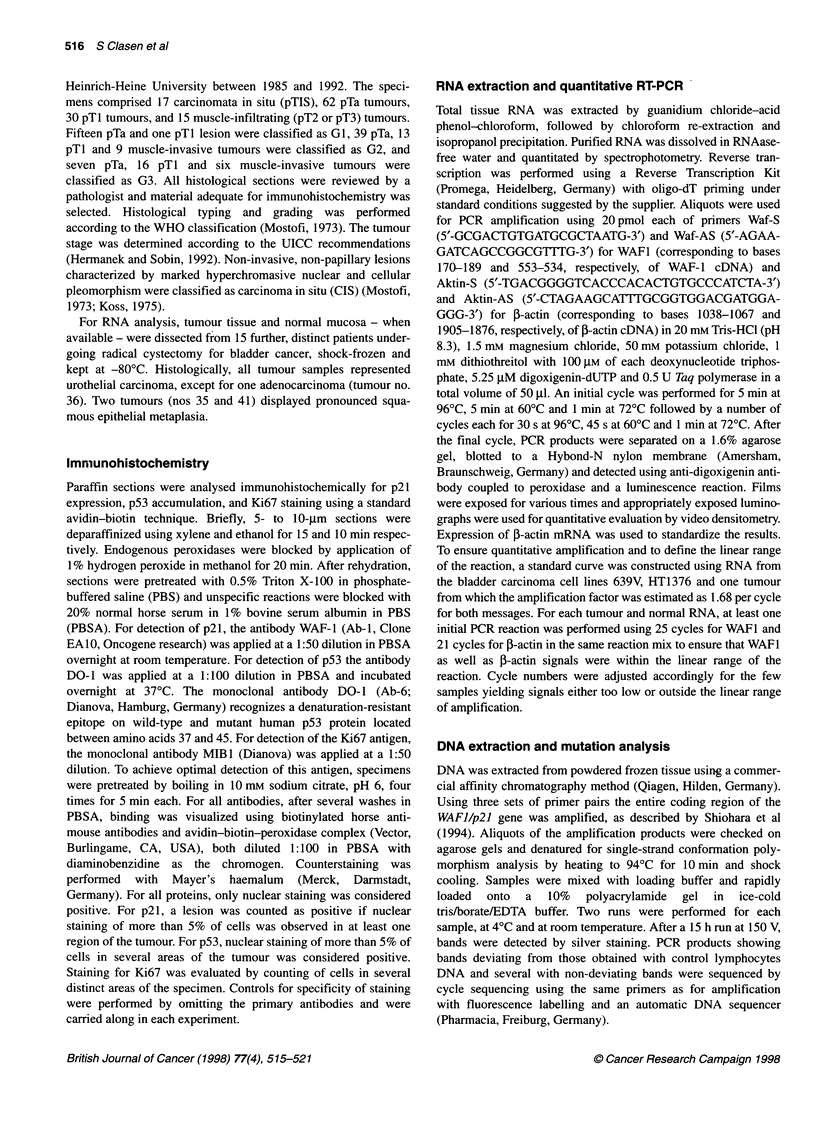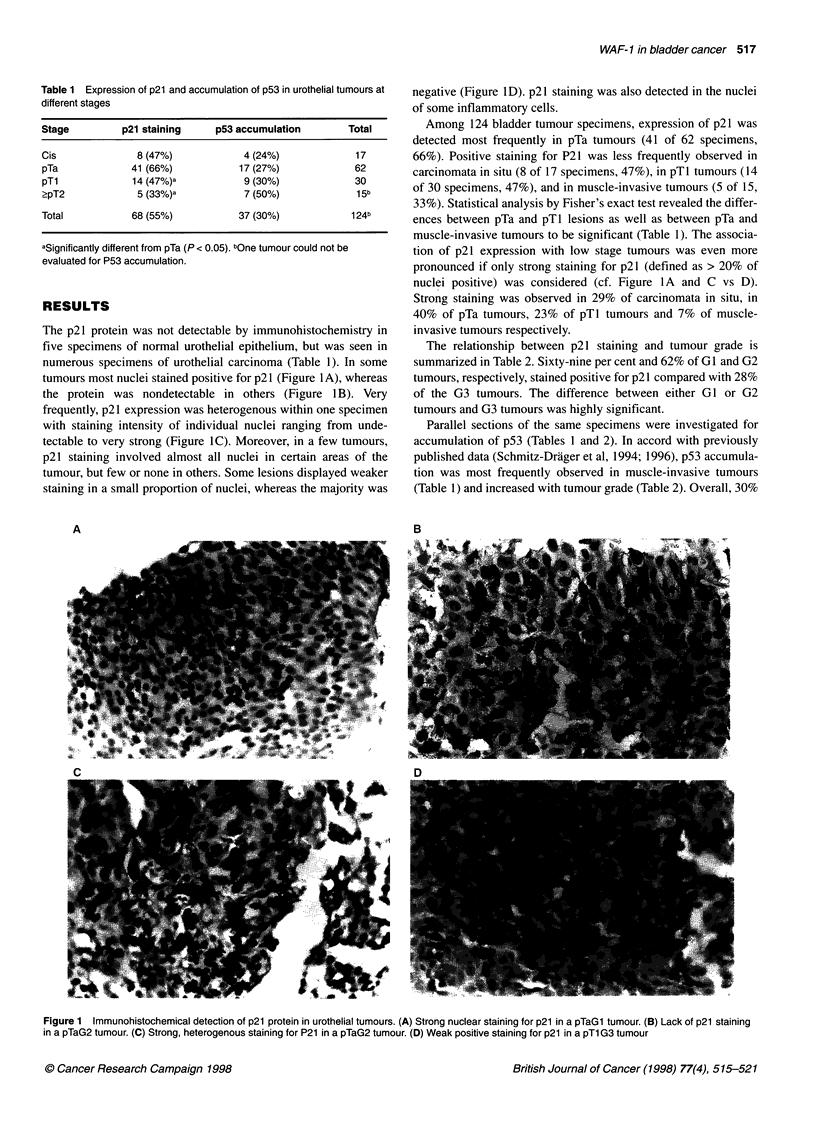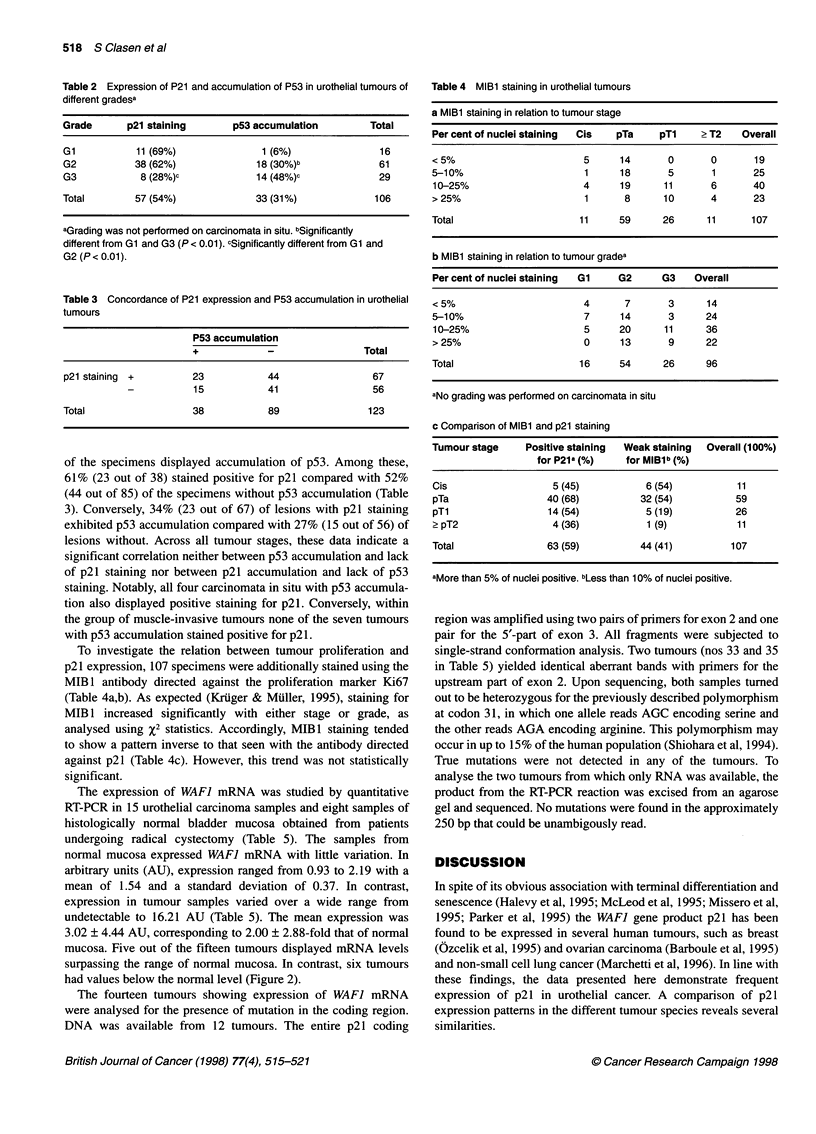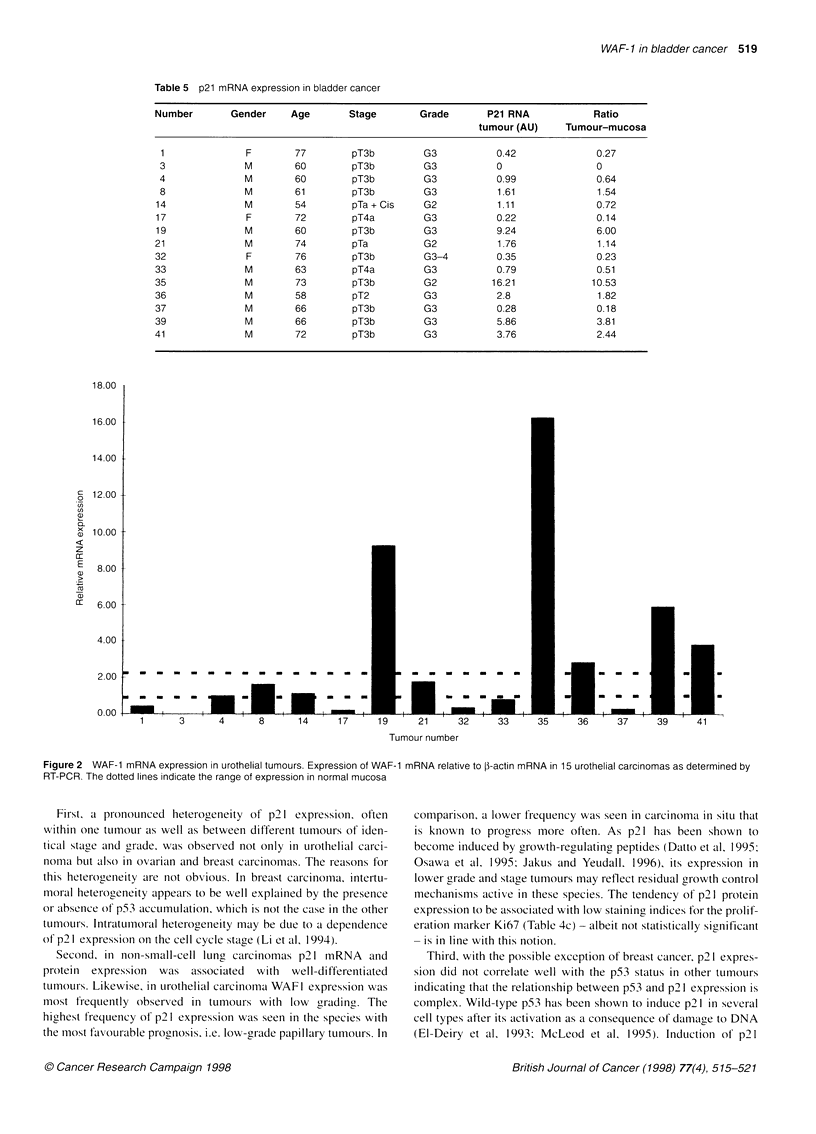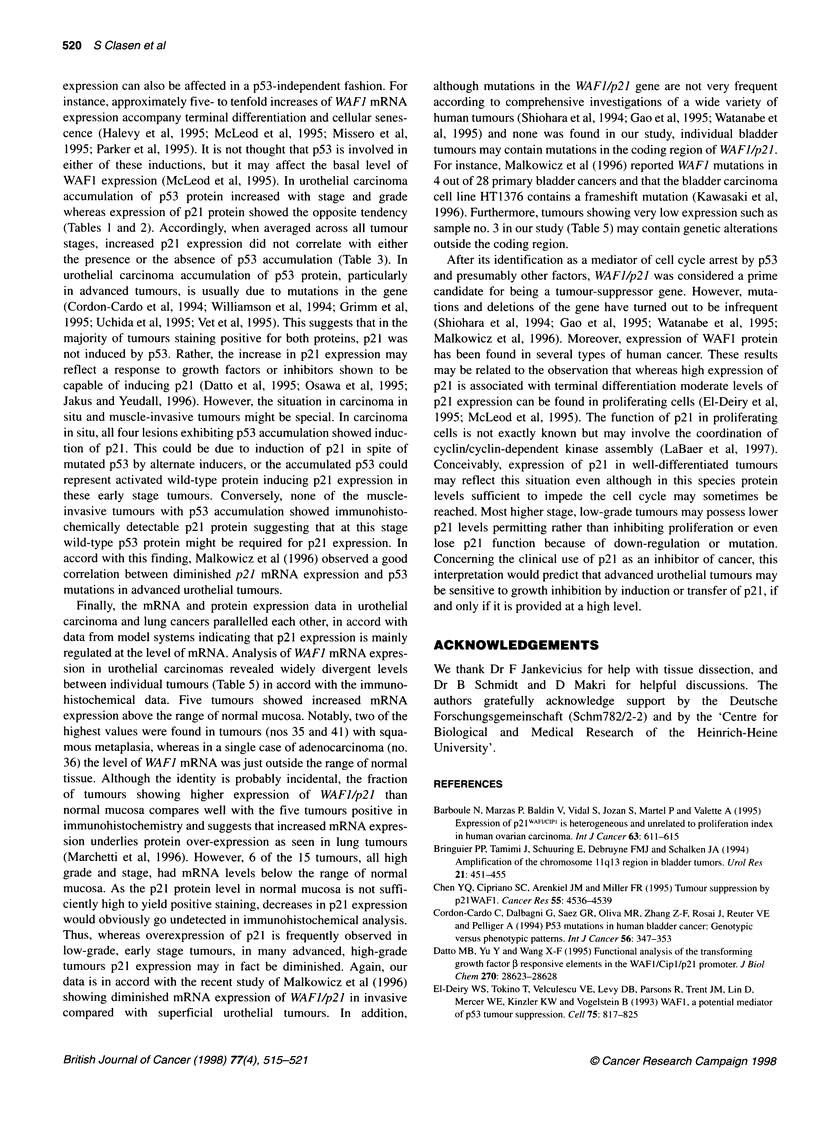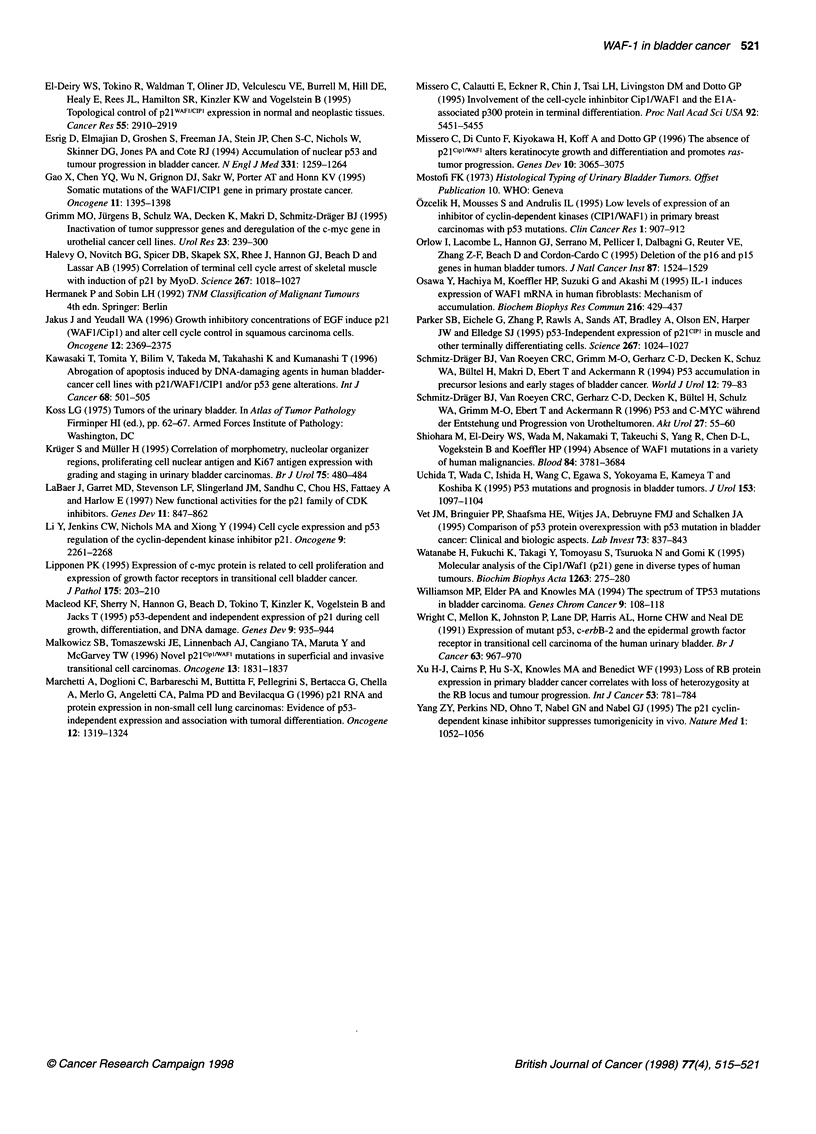# Frequent and heterogeneous expression of cyclin-dependent kinase inhibitor WAF1/p21 protein and mRNA in urothelial carcinoma.

**DOI:** 10.1038/bjc.1998.84

**Published:** 1998-02

**Authors:** S. Clasen, W. A. Schulz, C. D. Gerharz, M. O. Grimm, F. Christoph, B. J. Schmitz-DrÃ¤ger

**Affiliations:** Urologische Klinik, Heinrich-Heine-UniversitÃ¤t, DÃ¼sseldorf, Germany.

## Abstract

**Images:**


British Journal of Cancer (1998) 77(4), 515–521
© 1998 Cancer Research Campaign

### Frequent and heterogenous expression of cyclin-dependent kinase inhibitor WAF1/p21 protein and mRNA in urothelial carcinoma

**S Clasen[1], WA Schulz[1,2], C-D Gerharz[3], M-O Grimm[1], F Christoph[1] and BJ Schmitz-Dräger[1,2]**

[1]Urologische Klinik, [2]Biologisch-Medizinisches Forschungszentrum and [3]Institut für Pathologie, Heinrich-Heine-Universität, Moorenstr. 5, 40225 Düsseldorf, Germany

**Summary** The inhibitor of cyclin-dependent kinases WAF1/p21 has been shown to mediate cell cycle arrest by p53 and other factors. We have studied its expression in urothelial carcinoma. Immunohistochemistry of paraffin-embedded tissues revealed no detectable p21 protein in normal mucosa, whereas 8 of 17 (47%) carcinomata in situ, 41 of 62 (66%) pTa, 14 of 30 (47%) pT1 and 5 of 15 (33%) muscle-invasive tumours stained positive, usually with a heterogeneous pattern. Expression of p21 was associated with low grade tumours. In contrast, the frequency of p53 accumulation increased with grade and stage as did the frequency of staining for the proliferation marker Ki67. The level of WAF1 mRNA was determined relative to β-actin mRNA by quantitative reverse transcriptase polymerase chain reaction (RT-PCR) in 15 freshly frozen invasive tumours. In eight samples obtained from normal bladder mucosa, the values ranged from 0.93 to 2.19 arbitrary units (AU) (mean 1.54 ± 0.37 AU), but varied widely from non-detectable to 16.21 AU (mean 3.02 ± 4.44 AU) in the tumour specimens. In accord with the immunohistochemical findings, *WAF1* mRNA expression was elevated over the range found in normal mucosa in 5 of 15 advanced tumours. In addition, RNA analysis revealed a decrease in expression in six tumours. No mutations were observed in the *WAF1/p21* gene in these tumours, but two were heterozygous for the codon 31 polymorphism. These data indicate that p21 is frequently expressed in superficial, well differentiated urothelial carcinomas, but less often in muscle-invasive urothelial carcinomas, irrespective of their p53 status. The expression of p21 and its prevalence in low-stage tumours may reflect residual growth-regulatory influences potentially impeding but not necessarily inhibiting tumour development.

**Keywords:** p53; tumour suppressor; immunohistochemistry; quantitative reverse transcription polymerase chain reaction; cell cycle

Mutations and deregulation of genes involved in cell cycle control appear to play a central role in the development of urothelial carcinoma. Genes involved in cell cycle control and affected in urothelial carcinoma comprise *MTS1/p16* and *MTS2/p15* encoding inhibitors of cyclin-dependent kinases (CDKs) (Orlow et al, 1995), cyclin D1 (Bringuier et al, 1994), RB (Xu et al, 1993) and MYC (Lipponen, 1995; Schmitz-Dräger et al, 1996). In addition, depending on stage and grade, a considerable fraction of urothelial carcinomas display accumulation of p53 protein usually due to point mutations in the gene (Wright et al, 1991; Cordon-Cardo et al, 1994; Esrig et al, 1994; Schmitz-Dräger et al, 1994; Williamson et al, 1994; Uchida et al, 1995; Vet et al, 1995).

The wild-type p53 protein is capable of arresting the cell cycle in response to DNA damage by inducing a further CDK inhibitor WAF1/p21 (El-Deiry et al, 1993). Increased expression of p21 is also observed in some cell types undergoing terminal differentiation or senescence and may contribute to these processes (Halevy et al, 1995; McLeod et al, 1995; Missero et al, 1995; Parker et al, 1995). Although over-expression of p21 has been shown to suppress tumour formation (Chen et al, 1995; Yang et al, 1995) and the absence of WAF1/p21 facilitates tumour formation in several animal tumour models (El-Deiry et al, 1995; Missero et al, 1996), surveys on a variety of human tumours have revealed the *WAF1/p21* gene to be infrequently mutated (Shiohara et al, 1994; Gao et al, 1995; Watanabe et al, 1995). Instead, expression of p21 has been found in human tumours of different origin (Barboule et al, 1995; Özcelik et al, 1995; Marchetti et al, 1996).

In urothelial carcinoma, *WAF1/p21* mRNA has been shown to be expressed by reverse transcriptase polymerase chain reactions (RT-PCR). Among 28 tumours, four contained mutations in the gene (Malkowicz et al, 1996). Here, we report on the expression of p21 in urothelial carcinoma using immunohistochemistry to detect expression of the protein through several stages of tumour development. In addition, p53 protein accumulation was compared in the same tumour specimens to determine whether induction of p21 might be mediated by p53 and the MIB1 antibody detecting the Ki67 antigene was used to investigate the proliferation status. Quantitative RT-PCR was used to measure *WAF1* mRNA levels in advanced tumours, and these tumours were screened for mutations in the *WAF1* gene.

*Received 7 January 1997*
*Revised 7 July 1997*
*Accepted 21 August 1997*

*Correspondence to:* WA Schulz

### MATERIALS AND METHODS

### Patients and specimens

For immunohistochemistry, archival paraffin-embedded tumour specimens were derived from patients treated for primary or recurrent bladder tumours at the Department of Urology of the

Heinrich-Heine University between 1985 and 1992. The specimens comprised 17 carcinomata in situ (pTIS), 62 pTa tumours, 30 pT1 tumours, and 15 muscle-infiltrating (pT2 or pT3) tumours. Fifteen pTa and one pT1 lesion were classified as G1, 39 pTa, 13 pT1 and 9 muscle-invasive tumours were classified as G2, and seven pTa, 16 pT1 and six muscle-invasive tumours were classified as G3. All histological sections were reviewed by a pathologist and material adequate for immunohistochemistry was selected. Histological typing and grading was performed according to the WHO classification (Mostofi, 1973). The tumour stage was determined according to the UICC recommendations (Hermanek and Sobin, 1992). Non-invasive, non-papillary lesions characterized by marked hyperchromasive nuclear and cellular pleomorphism were classified as carcinoma in situ (CIS) (Mostofi, 1973; Koss, 1975).

For RNA analysis, tumour tissue and normal mucosa – when available – were dissected from 15 further, distinct patients undergoing radical cystectomy for bladder cancer, shock-frozen and kept at −80°C. Histologically, all tumour samples represented urothelial carcinoma, except for one adenocarcinoma (tumour no. 36). Two tumours (nos 35 and 41) displayed pronounced squamous epithelial metaplasia.

### Immunohistochemistry

Paraffin sections were analysed immunohistochemically for p21 expression, p53 accumulation, and Ki67 staining using a standard avidin–biotin technique. Briefly, 5- to 10-μm sections were deparaffinized using xylene and ethanol for 15 and 10 min respectively. Endogenous peroxidases were blocked by application of 1% hydrogen peroxide in methanol for 20 min. After rehydration, sections were pretreated with 0.5% Triton X-100 in phosphate-buffered saline (PBS) and unspecific reactions were blocked with 20% normal horse serum in 1% bovine serum albumin in PBS (PBSA). For detection of p21, the antibody WAF-1 (Ab-1, Clone EA10, Oncogene research) was applied at a 1:50 dilution in PBSA overnight at room temperature. For detection of p53 the antibody DO-1 was applied at a 1:100 dilution in PBSA and incubated overnight at 37°C. The monoclonal antibody DO-1 (Ab-6; Dianova, Hamburg, Germany) recognizes a denaturation-resistant epitope on wild-type and mutant human p53 protein located between amino acids 37 and 45. For detection of the Ki67 antigen, the monoclonal antibody MIB1 (Dianova) was applied at a 1:50 dilution. To achieve optimal detection of this antigen, specimens were pretreated by boiling in 10 mM sodium citrate, pH 6, four times for 5 min each. For all antibodies, after several washes in PBSA, binding was visualized using biotinylated horse anti-mouse antibodies and avidin–biotin–peroxidase complex (Vector, Burlingame, CA, USA), both diluted 1:100 in PBSA with diaminobenzidine as the chromogen. Counterstaining was performed with Mayer's haemalum (Merck, Darmstadt, Germany). For all proteins, only nuclear staining was considered positive. For p21, a lesion was counted as positive if nuclear staining of more than 5% of cells was observed in at least one region of the tumour. For p53, nuclear staining of more than 5% of cells in several areas of the tumour was considered positive. Staining for Ki67 was evaluated by counting of cells in several distinct areas of the specimen. Controls for specificity of staining were performed by omitting the primary antibodies and were carried along in each experiment.

### RNA extraction and quantitative RT-PCR

Total tissue RNA was extracted by guanidium chloride–acid phenol–chloroform, followed by chloroform re-extraction and isopropanol precipitation. Purified RNA was dissolved in RNAase-free water and quantitated by spectrophotometry. Reverse transcription was performed using a Reverse Transcription Kit (Promega, Heidelberg, Germany) with oligo-dT priming under standard conditions suggested by the supplier. Aliquots were used for PCR amplification using 20 pmol each of primers Waf-S (5′-GCGACTGTGATGCGCTAATG-3′) and Waf-AS (5′-AGAAGATCAGCCGGCGTTTG-3′) for WAF1 (corresponding to bases 170–189 and 553–534, respectively, of WAF-1 cDNA) and Aktin-S (5′-TGACGGGGTCACCCACACTGTGCCCATCTA-3′) and Aktin-AS (5′-CTAGAAGCATTTGCGGTGGACGATGGAGGG-3′) for β-actin (corresponding to bases 1038–1067 and 1905–1876, respectively, of β-actin cDNA) in 20 mM Tris-HCl (pH 8.3), 1.5 mM magnesium chloride, 50 mM potassium chloride, 1 mM dithiothreitol with 100 μM of each deoxynucleotide triphosphate, 5.25 μM digoxigenin-dUTP and 0.5 U *Taq* polymerase in a total volume of 50 μl. An initial cycle was performed for 5 min at 96°C, 5 min at 60°C and 1 min at 72°C followed by a number of cycles each for 30 s at 96°C, 45 s at 60°C and 1 min at 72°C. After the final cycle, PCR products were separated on a 1.6% agarose gel, blotted to a Hybond-N nylon membrane (Amersham, Braunschweig, Germany) and detected using anti-digoxigenin antibody coupled to peroxidase and a luminescence reaction. Films were exposed for various times and appropriately exposed luminographs were used for quantitative evaluation by video densitometry. Expression of β-actin mRNA was used to standardize the results. To ensure quantitative amplification and to define the linear range of the reaction, a standard curve was constructed using RNA from the bladder carcinoma cell lines 639V, HT1376 and one tumour from which the amplification factor was estimated as 1.68 per cycle for both messages. For each tumour and normal RNA, at least one initial PCR reaction was performed using 25 cycles for WAF1 and 21 cycles for β-actin in the same reaction mix to ensure that WAF1 as well as β-actin signals were within the linear range of the reaction. Cycle numbers were adjusted accordingly for the few samples yielding signals either too low or outside the linear range of amplification.

### DNA extraction and mutation analysis

DNA was extracted from powdered frozen tissue using a commercial affinity chromatography method (Qiagen, Hilden, Germany). Using three sets of primer pairs the entire coding region of the *WAF1/p21* gene was amplified, as described by Shiohara et al (1994). Aliquots of the amplification products were checked on agarose gels and denatured for single-strand conformation polymorphism analysis by heating to 94°C for 10 min and shock cooling. Samples were mixed with loading buffer and rapidly loaded onto a 10% polyacrylamide gel in ice-cold tris/borate/EDTA buffer. Two runs were performed for each sample, at 4°C and at room temperature. After a 15 h run at 150 V, bands were detected by silver staining. PCR products showing bands deviating from those obtained with control lymphocytes DNA and several with non-deviating bands were sequenced by cycle sequencing using the same primers as for amplification with fluorescence labelling and an automatic DNA sequencer (Pharmacia, Freiburg, Germany).

© Cancer Research Campaign 1998

**Table 1** Expression of p21 and accumulation of p53 in urothelial tumours at different stages

| Stage | p21 staining | p53 accumulation | Total |
|---|---|---|---|
| Cis | 8 (47%) | 4 (24%) | 17 |
| pTa | 41 (66%) | 17 (27%) | 62 |
| pT1 | 14 (47%)[a] | 9 (30%) | 30 |
| ≥pT2 | 5 (33%)[a] | 7 (50%) | 15[b] |
| Total | 68 (55%) | 37 (30%) | 124[b] |

[a]Significantly different from pTa ($P < 0.05$). [b]One tumour could not be evaluated for P53 accumulation.

### RESULTS

The p21 protein was not detectable by immunohistochemistry in five specimens of normal urothelial epithelium, but was seen in numerous specimens of urothelial carcinoma (Table 1). In some tumours most nuclei stained positive for p21 (Figure 1A), whereas the protein was nondetectable in others (Figure 1B). Very frequently, p21 expression was heterogenous within one specimen with staining intensity of individual nuclei ranging from undetectable to very strong (Figure 1C). Moreover, in a few tumours, p21 staining involved almost all nuclei in certain areas of the tumour, but few or none in others. Some lesions displayed weaker staining in a small proportion of nuclei, whereas the majority was negative (Figure 1D). p21 staining was also detected in the nuclei of some inflammatory cells.

Among 124 bladder tumour specimens, expression of p21 was detected most frequently in pTa tumours (41 of 62 specimens, 66%). Positive staining for P21 was less frequently observed in carcinomata in situ (8 of 17 specimens, 47%), in pT1 tumours (14 of 30 specimens, 47%), and in muscle-invasive tumours (5 of 15, 33%). Statistical analysis by Fisher's exact test revealed the differences between pTa and pT1 lesions as well as between pTa and muscle-invasive tumours to be significant (Table 1). The association of p21 expression with low stage tumours was even more pronounced if only strong staining for p21 (defined as > 20% of nuclei positive) was considered (cf. Figure 1A and C vs D). Strong staining was observed in 29% of carcinomata in situ, in 40% of pTa tumours, 23% of pT1 tumours and 7% of muscle-invasive tumours respectively.

The relationship between p21 staining and tumour grade is summarized in Table 2. Sixty-nine per cent and 62% of G1 and G2 tumours, respectively, stained positive for p21 compared with 28% of the G3 tumours. The difference between either G1 or G2 tumours and G3 tumours was highly significant.

Parallel sections of the same specimens were investigated for accumulation of p53 (Tables 1 and 2). In accord with previously published data (Schmitz-Dräger et al, 1994; 1996), p53 accumulation was most frequently observed in muscle-invasive tumours (Table 1) and increased with tumour grade (Table 2). Overall, 30%

**Figure 1** Immunohistochemical detection of p21 protein in urothelial tumours. (**A**) Strong nuclear staining for p21 in a pTaG1 tumour. (**B**) Lack of p21 staining in a pTaG2 tumour. (**C**) Strong, heterogenous staining for P21 in a pTaG2 tumour. (**D**) Weak positive staining for p21 in a pT1G3 tumour

© Cancer Research Campaign 1998

**Table 2** Expression of P21 and accumulation of P53 in urothelial tumours of different grades[a]

| Grade | p21 staining | p53 accumulation | Total |
|---|---|---|---|
| G1 | 11 (69%) | 1 (6%) | 16 |
| G2 | 38 (62%) | 18 (30%)[b] | 61 |
| G3 | 8 (28%)[c] | 14 (48%)[c] | 29 |
| Total | 57 (54%) | 33 (31%) | 106 |

[a]Grading was not performed on carcinomata in situ. [b]Significantly different from G1 and G3 ($P < 0.01$). [c]Significantly different from G1 and G2 ($P < 0.01$).

**Table 3** Concordance of P21 expression and P53 accumulation in urothelial tumours

| | | P53 accumulation | | |
|---|---|---|---|---|
| | | + | – | Total |
| p21 staining | + | 23 | 44 | 67 |
| | – | 15 | 41 | 56 |
| Total | | 38 | 89 | 123 |

of the specimens displayed accumulation of p53. Among these, 61% (23 out of 38) stained positive for p21 compared with 52% (44 out of 85) of the specimens without p53 accumulation (Table 3). Conversely, 34% (23 out of 67) of lesions with p21 staining exhibited p53 accumulation compared with 27% (15 out of 56) of lesions without. Across all tumour stages, these data indicate a significant correlation neither between p53 accumulation and lack of p21 staining nor between p21 accumulation and lack of p53 staining. Notably, all four carcinomata in situ with p53 accumulation also displayed positive staining for p21. Conversely, within the group of muscle-invasive tumours none of the seven tumours with p53 accumulation stained positive for p21.

To investigate the relation between tumour proliferation and p21 expression, 107 specimens were additionally stained using the MIB1 antibody directed against the proliferation marker Ki67 (Table 4a,b). As expected (Krüger & Müller, 1995), staining for MIB1 increased significantly with either stage or grade, as analysed using $\chi^2$ statistics. Accordingly, MIB1 staining tended to show a pattern inverse to that seen with the antibody directed against p21 (Table 4c). However, this trend was not statistically significant.

The expression of *WAF1* mRNA was studied by quantitative RT-PCR in 15 urothelial carcinoma samples and eight samples of histologically normal bladder mucosa obtained from patients undergoing radical cystectomy (Table 5). The samples from normal mucosa expressed *WAF1* mRNA with little variation. In arbitrary units (AU), expression ranged from 0.93 to 2.19 with a mean of 1.54 and a standard deviation of 0.37. In contrast, expression in tumour samples varied over a wide range from undetectable to 16.21 AU (Table 5). The mean expression was 3.02 ± 4.44 AU, corresponding to 2.00 ± 2.88-fold that of normal mucosa. Five out of the fifteen tumours displayed mRNA levels surpassing the range of normal mucosa. In contrast, six tumours had values below the normal level (Figure 2).

The fourteen tumours showing expression of *WAF1* mRNA were analysed for the presence of mutation in the coding region. DNA was available from 12 tumours. The entire p21 coding

**Table 4** MIB1 staining in urothelial tumours

**a** MIB1 staining in relation to tumour stage

| Per cent of nuclei staining | Cis | pTa | pT1 | ≥ T2 | Overall |
|---|---|---|---|---|---|
| < 5% | 5 | 14 | 0 | 0 | 19 |
| 5–10% | 1 | 18 | 5 | 1 | 25 |
| 10–25% | 4 | 19 | 11 | 6 | 40 |
| > 25% | 1 | 8 | 10 | 4 | 23 |
| Total | 11 | 59 | 26 | 11 | 107 |

**b** MIB1 staining in relation to tumour grade[a]

| Per cent of nuclei staining | G1 | G2 | G3 | Overall |
|---|---|---|---|---|
| < 5% | 4 | 7 | 3 | 14 |
| 5–10% | 7 | 14 | 3 | 24 |
| 10–25% | 5 | 20 | 11 | 36 |
| > 25% | 0 | 13 | 9 | 22 |
| Total | 16 | 54 | 26 | 96 |

[a]No grading was performed on carcinomata in situ

**c** Comparison of MIB1 and p21 staining

| Tumour stage | Positive staining for P21[a] (%) | Weak staining for MIB1[b] (%) | Overall (100%) |
|---|---|---|---|
| Cis | 5 (45) | 6 (54) | 11 |
| pTa | 40 (68) | 32 (54) | 59 |
| pT1 | 14 (54) | 5 (19) | 26 |
| ≥ pT2 | 4 (36) | 1 (9) | 11 |
| Total | 63 (59) | 44 (41) | 107 |

[a]More than 5% of nuclei positive. [b]Less than 10% of nuclei positive.

region was amplified using two pairs of primers for exon 2 and one pair for the 5′-part of exon 3. All fragments were subjected to single-strand conformation analysis. Two tumours (nos 33 and 35 in Table 5) yielded identical aberrant bands with primers for the upstream part of exon 2. Upon sequencing, both samples turned out to be heterozygous for the previously described polymorphism at codon 31, in which one allele reads AGC encoding serine and the other reads AGA encoding arginine. This polymorphism may occur in up to 15% of the human population (Shiohara et al, 1994). True mutations were not detected in any of the tumours. To analyse the two tumours from which only RNA was available, the product from the RT-PCR reaction was excised from an agarose gel and sequenced. No mutations were found in the approximately 250 bp that could be unambigously read.

### DISCUSSION

In spite of its obvious association with terminal differentiation and senescence (Halevy et al, 1995; McLeod et al, 1995; Missero et al, 1995; Parker et al, 1995) the *WAF1* gene product p21 has been found to be expressed in several human tumours, such as breast (Özcelik et al, 1995) and ovarian carcinoma (Barboule et al, 1995) and non-small cell lung cancer (Marchetti et al, 1996). In line with these findings, the data presented here demonstrate frequent expression of p21 in urothelial cancer. A comparison of p21 expression patterns in the different tumour species reveals several similarities.

 © Cancer Research Campaign 1998

**Table 5** p21 mRNA expression in bladder cancer

| Number | Gender | Age | Stage | Grade | P21 RNA tumour (AU) | Ratio Tumour–mucosa |
|---|---|---|---|---|---|---|
| 1 | F | 77 | pT3b | G3 | 0.42 | 0.27 |
| 3 | M | 60 | pT3b | G3 | 0 | 0 |
| 4 | M | 60 | pT3b | G3 | 0.99 | 0.64 |
| 8 | M | 61 | pT3b | G3 | 1.61 | 1.54 |
| 14 | M | 54 | pTa + Cis | G2 | 1.11 | 0.72 |
| 17 | F | 72 | pT4a | G3 | 0.22 | 0.14 |
| 19 | M | 60 | pT3b | G3 | 9.24 | 6.00 |
| 21 | M | 74 | pTa | G2 | 1.76 | 1.14 |
| 32 | F | 76 | pT3b | G3–4 | 0.35 | 0.23 |
| 33 | M | 63 | pT4a | G3 | 0.79 | 0.51 |
| 35 | M | 73 | pT3b | G2 | 16.21 | 10.53 |
| 36 | M | 58 | pT2 | G3 | 2.8 | 1.82 |
| 37 | M | 66 | pT3b | G3 | 0.28 | 0.18 |
| 39 | M | 66 | pT3b | G3 | 5.86 | 3.81 |
| 41 | M | 72 | pT3b | G3 | 3.76 | 2.44 |

**Figure 2** WAF-1 mRNA expression in urothelial tumours. Expression of WAF-1 mRNA relative to β-actin mRNA in 15 urothelial carcinomas as determined by RT-PCR. The dotted lines indicate the range of expression in normal mucosa

First, a pronounced heterogeneity of p21 expression, often within one tumour as well as between different tumours of identical stage and grade, was observed not only in urothelial carcinoma but also in ovarian and breast carcinomas. The reasons for this heterogeneity are not obvious. In breast carcinoma, intertumoral heterogeneity appears to be well explained by the presence or absence of p53 accumulation, which is not the case in the other tumours. Intratumoral heterogeneity may be due to a dependence of p21 expression on the cell cycle stage (Li et al, 1994).

Second, in non-small-cell lung carcinomas p21 mRNA and protein expression was associated with well-differentiated tumours. Likewise, in urothelial carcinoma WAF1 expression was most frequently observed in tumours with low grading. The highest frequency of p21 expression was seen in the species with the most favourable prognosis, i.e. low-grade papillary tumours. In comparison, a lower frequency was seen in carcinoma in situ that is known to progress more often. As p21 has been shown to become induced by growth-regulating peptides (Datto et al, 1995; Osawa et al, 1995; Jakus and Yeudall, 1996), its expression in lower grade and stage tumours may reflect residual growth control mechanisms active in these species. The tendency of p21 protein expression to be associated with low staining indices for the proliferation marker Ki67 (Table 4c) – albeit not statistically significant – is in line with this notion.

Third, with the possible exception of breast cancer, p21 expression did not correlate well with the p53 status in other tumours indicating that the relationship between p53 and p21 expression is complex. Wild-type p53 has been shown to induce p21 in several cell types after its activation as a consequence of damage to DNA (El-Deiry et al, 1993; McLeod et al, 1995). Induction of p21

expression can also be affected in a p53-independent fashion. For instance, approximately five- to tenfold increases of *WAF1* mRNA expression accompany terminal differentiation and cellular senescence (Halevy et al, 1995; McLeod et al, 1995; Missero et al, 1995; Parker et al, 1995). It is not thought that p53 is involved in either of these inductions, but it may affect the basal level of WAF1 expression (McLeod et al, 1995). In urothelial carcinoma accumulation of p53 protein increased with stage and grade whereas expression of p21 protein showed the opposite tendency (Tables 1 and 2). Accordingly, when averaged across all tumour stages, increased p21 expression did not correlate with either the presence or the absence of p53 accumulation (Table 3). In urothelial carcinoma accumulation of p53 protein, particularly in advanced tumours, is usually due to mutations in the gene (Cordon-Cardo et al, 1994; Williamson et al, 1994; Grimm et al, 1995; Uchida et al, 1995; Vet et al, 1995). This suggests that in the majority of tumours staining positive for both proteins, p21 was not induced by p53. Rather, the increase in p21 expression may reflect a response to growth factors or inhibitors shown to be capable of inducing p21 (Datto et al, 1995; Osawa et al, 1995; Jakus and Yeudall, 1996). However, the situation in carcinoma in situ and muscle-invasive tumours might be special. In carcinoma in situ, all four lesions exhibiting p53 accumulation showed induction of p21. This could be due to induction of p21 in spite of mutated p53 by alternate inducers, or the accumulated p53 could represent activated wild-type protein inducing p21 expression in these early stage tumours. Conversely, none of the muscle-invasive tumours with p53 accumulation showed immunohistochemically detectable p21 protein suggesting that at this stage wild-type p53 protein might be required for p21 expression. In accord with this finding, Malkowicz et al (1996) observed a good correlation between diminished *p21* mRNA expression and p53 mutations in advanced urothelial tumours.

Finally, the mRNA and protein expression data in urothelial carcinoma and lung cancers parallelled each other, in accord with data from model systems indicating that p21 expression is mainly regulated at the level of mRNA. Analysis of *WAF1* mRNA expression in urothelial carcinomas revealed widely divergent levels between individual tumours (Table 5) in accord with the immunohistochemical data. Five tumours showed increased mRNA expression above the range of normal mucosa. Notably, two of the highest values were found in tumours (nos 35 and 41) with squamous metaplasia, whereas in a single case of adenocarcinoma (no. 36) the level of *WAF1* mRNA was just outside the range of normal tissue. Although the identity is probably incidental, the fraction of tumours showing higher expression of *WAF1/p21* than normal mucosa compares well with the five tumours positive in immunohistochemistry and suggests that increased mRNA expression underlies protein over-expression as seen in lung tumours (Marchetti et al, 1996). However, 6 of the 15 tumours, all high grade and stage, had mRNA levels below the range of normal mucosa. As the p21 protein level in normal mucosa is not sufficiently high to yield positive staining, decreases in p21 expression would obviously go undetected in immunohistochemical analysis. Thus, whereas overexpression of p21 is frequently observed in low-grade, early stage tumours, in many advanced, high-grade tumours p21 expression may in fact be diminished. Again, our data is in accord with the recent study of Malkowicz et al (1996) showing diminished mRNA expression of *WAF1/p21* in invasive compared with superficial urothelial tumours. In addition, although mutations in the *WAF1/p21* gene are not very frequent according to comprehensive investigations of a wide variety of human tumours (Shiohara et al, 1994; Gao et al, 1995; Watanabe et al, 1995) and none was found in our study, individual bladder tumours may contain mutations in the coding region of *WAF1/p21*. For instance, Malkowicz et al (1996) reported *WAF1* mutations in 4 out of 28 primary bladder cancers and that the bladder carcinoma cell line HT1376 contains a frameshift mutation (Kawasaki et al, 1996). Furthermore, tumours showing very low expression such as sample no. 3 in our study (Table 5) may contain genetic alterations outside the coding region.

After its identification as a mediator of cell cycle arrest by p53 and presumably other factors, *WAF1/p21* was considered a prime candidate for being a tumour-suppressor gene. However, mutations and deletions of the gene have turned out to be infrequent (Shiohara et al, 1994; Gao et al, 1995; Watanabe et al, 1995; Malkowicz et al, 1996). Moreover, expression of WAF1 protein has been found in several types of human cancer. These results may be related to the observation that whereas high expression of p21 is associated with terminal differentiation moderate levels of p21 expression can be found in proliferating cells (El-Deiry et al, 1995; McLeod et al, 1995). The function of p21 in proliferating cells is not exactly known but may involve the coordination of cyclin/cyclin-dependent kinase assembly (LaBaer et al, 1997). Conceivably, expression of p21 in well-differentiated tumours may reflect this situation even although in this species protein levels sufficient to impede the cell cycle may sometimes be reached. Most higher stage, low-grade tumours may possess lower p21 levels permitting rather than inhibiting proliferation or even lose p21 function because of down-regulation or mutation. Concerning the clinical use of p21 as an inhibitor of cancer, this interpretation would predict that advanced urothelial tumours may be sensitive to growth inhibition by induction or transfer of p21, if and only if it is provided at a high level.

### ACKNOWLEDGEMENTS

We thank Dr F Jankevicius for help with tissue dissection, and Dr B Schmidt and D Makri for helpful discussions. The authors gratefully acknowledge support by the Deutsche Forschungsgemeinschaft (Schm782/2-2) and by the 'Centre for Biological and Medical Research of the Heinrich-Heine University'.

### REFERENCES

Barboule N, Marzas P, Baldin V, Vidal S, Jozan S, Martel P and Valette A (1995) Expression of p21$^{WAF1/CIP1}$ is heterogeneous and unrelated to proliferation index in human ovarian carcinoma. *Int J Cancer* **63**: 611–615

Bringuier PP, Tamimi J, Schuuring E, Debruyne FMJ and Schalken JA (1994) Amplification of the chromosome 11q13 region in bladder tumors. *Urol Res* **21**: 451–455

Chen YQ, Cipriano SC, Arenkiel JM and Miller FR (1995) Tumour suppression by p21WAF1. *Cancer Res* **55**: 4536–4539

Cordon-Cardo C, Dalbagni G, Saez GR, Oliva MR, Zhang Z-F, Rosai J, Reuter VE and Pelliger A (1994) P53 mutations in human bladder cancer: Genotypic versus phenotypic patterns. *Int J Cancer* **56**: 347–353

Datto MB, Yu Y and Wang X-F (1995) Functional analysis of the transforming growth factor β responsive elements in the WAF1/Cip1/p21 promoter. *J Biol Chem* **270**: 28623–28628

El-Deiry WS, Tokino T, Velculescu VE, Levy DB, Parsons R, Trent JM, Lin D, Mercer WE, Kinzler KW and Vogelstein B (1993) WAF1, a potential mediator of p53 tumour suppression. *Cell* **75**: 817–825

© Cancer Research Campaign 1998

El-Deiry WS, Tokino R, Waldman T, Oliner JD, Velculescu VE, Burrell M, Hill DE, Healy E, Rees JL, Hamilton SR, Kinzler KW and Vogelstein B (1995) Topological control of p21[WAF1/CIP1] expression in normal and neoplastic tissues. *Cancer Res* **55**: 2910–2919

Esrig D, Elmajian D, Groshen S, Freeman JA, Stein JP, Chen S-C, Nichols W, Skinner DG, Jones PA and Cote RJ (1994) Accumulation of nuclear p53 and tumour progression in bladder cancer. *N Engl J Med* **331**: 1259–1264

Gao X, Chen YQ, Wu N, Grignon DJ, Sakr W, Porter AT and Honn KV (1995) Somatic mutations of the WAF1/CIP1 gene in primary prostate cancer. *Oncogene* **11**: 1395–1398

Grimm MO, Jürgens B, Schulz WA, Decken K, Makri D, Schmitz-Dräger BJ (1995) Inactivation of tumor suppressor genes and deregulation of the c-myc gene in urothelial cancer cell lines. *Urol Res* **23**: 239–300

Halevy O, Novitch BG, Spicer DB, Skapek SX, Rhee J, Hannon GJ, Beach D and Lassar AB (1995) Correlation of terminal cell cycle arrest of skeletal muscle with induction of p21 by MyoD. *Science* **267**: 1018–1027

Hermanek P and Sobin LH (1992) *TNM Classification of Malignant Tumours* 4th edn. Springer: Berlin

Jakus J and Yeudall WA (1996) Growth inhibitory concentrations of EGF induce p21 (WAF1/Cip1) and alter cell cycle control in squamous carcinoma cells. *Oncogene* **12**: 2369–2375

Kawasaki T, Tomita Y, Bilim V, Takeda M, Takahashi K and Kumanashi T (1996) Abrogation of apoptosis induced by DNA-damaging agents in human bladder-cancer cell lines with p21/WAF1/CIP1 and/or p53 gene alterations. *Int J Cancer* **68**: 501–505

Koss LG (1975) Tumors of the urinary bladder. In *Atlas of Tumor Pathology* Firminper HI (ed.), pp. 62–67. Armed Forces Institute of Pathology: Washington, DC

Krüger S and Müller H (1995) Correlation of morphometry, nucleolar organizer regions, proliferating cell nuclear antigen and Ki67 antigen expression with grading and staging in urinary bladder carcinomas. *Br J Urol* **75**: 480–484

LaBaer J, Garret MD, Stevenson LF, Slingerland JM, Sandhu C, Chou HS, Fattaey A and Harlow E (1997) New functional activities for the p21 family of CDK inhibitors. *Genes Dev* **11**: 847–862

Li Y, Jenkins CW, Nichols MA and Xiong Y (1994) Cell cycle expression and p53 regulation of the cyclin-dependent kinase inhibitor p21. *Oncogene* **9**: 2261–2268

Lipponen PK (1995) Expression of c-myc protein is related to cell proliferation and expression of growth factor receptors in transitional cell bladder cancer. *J Pathol* **175**: 203–210

Macleod KF, Sherry N, Hannon G, Beach D, Tokino T, Kinzler K, Vogelstein B and Jacks T (1995) p53-dependent and independent expression of p21 during cell growth, differentiation, and DNA damage. *Genes Dev* **9**: 935–944

Malkowicz SB, Tomaszewski JE, Linnenbach AJ, Cangiano TA, Maruta Y and McGarvey TW (1996) Novel p21[Cip1/WAF1] mutations in superficial and invasive transitional cell carcinomas. *Oncogene* **13**: 1831–1837

Marchetti A, Doglioni C, Barbareschi M, Buttitta F, Pellegrini S, Bertacca G, Chella A, Merlo G, Angeletti CA, Palma PD and Bevilacqua G (1996) p21 RNA and protein expression in non-small cell lung carcinomas: Evidence of p53-independent expression and association with tumoral differentiation. *Oncogene* **12**: 1319–1324

Missero C, Calautti E, Eckner R, Chin J, Tsai LH, Livingston DM and Dotto GP (1995) Involvement of the cell-cycle inhinbitor Cip1/WAF1 and the E1A-associated p300 protein in terminal differentiation. *Proc Natl Acad Sci USA* **92**: 5451–5455

Missero C, Di Cunto F, Kiyokawa H, Koff A and Dotto GP (1996) The absence of p21[Cip1/WAF1] alters keratinocyte growth and differentiation and promotes *ras*-tumor progression. *Genes Dev* **10**: 3065–3075

Mostofi FK (1973) *Histological Typing of Urinary Bladder Tumors. Offset Publication* 10. WHO: Geneva

Özcelik H, Mousses S and Andrulis IL (1995) Low levels of expression of an inhibitor of cyclin-dependent kinases (CIP1/WAF1) in primary breast carcinomas with p53 mutations. *Clin Cancer Res* **1**: 907–912

Orlow I, Lacombe L, Hannon GJ, Serrano M, Pellicer I, Dalbagni G, Reuter VE, Zhang Z-F, Beach D and Cordon-Cardo C (1995) Deletion of the p16 and p15 genes in human bladder tumors. *J Natl Cancer Inst* **87**: 1524–1529

Osawa Y, Hachiya M, Koeffler HP, Suzuki G and Akashi M (1995) IL-1 induces expression of WAF1 mRNA in human fibroblasts: Mechanism of accumulation. *Biochem Biophys Res Commun* **216**: 429–437

Parker SB, Eichele G, Zhang P, Rawls A, Sands AT, Bradley A, Olson EN, Harper JW and Elledge SJ (1995) p53-Independent expression of p21[CIP1] in muscle and other terminally differentiating cells. *Science* **267**: 1024–1027

Schmitz-Dräger BJ, Van Roeyen CRC, Grimm M-O, Gerharz C-D, Decken K, Schuz WA, Bültel H, Makri D, Ebert T and Ackermann R (1994) P53 accumulation in precursor lesions and early stages of bladder cancer. *World J Urol* **12**: 79–83

Schmitz-Dräger BJ, Van Roeyen CRC, Gerharz C-D, Decken K, Bültel H, Schulz WA, Grimm M-O, Ebert T and Ackermann R (1996) P53 and C-MYC während der Entstehung und Progression von Urotheltumoren. *Akt Urol* **27**: 55–60

Shiohara M, El-Deiry WS, Wada M, Nakamaki T, Takeuchi S, Yang R, Chen D-L, Vogekstein B and Koeffler HP (1994) Absence of WAF1 mutations in a variety of human malignancies. *Blood* **84**: 3781–3684

Uchida T, Wada C, Ishida H, Wang C, Egawa S, Yokoyama E, Kameya T and Koshiba K (1995) P53 mutations and prognosis in bladder tumors. *J Urol* **153**: 1097–1104

Vet JM, Bringuier PP, Shaafsma HE, Witjes JA, Debruyne FMJ and Schalken JA (1995) Comparison of p53 protein overexpression with p53 mutation in bladder cancer: Clinical and biologic aspects. *Lab Invest* **73**: 837–843

Watanabe H, Fukuchi K, Takagi Y, Tomoyasu S, Tsuruoka N and Gomi K (1995) Molecular analysis of the Cip1/Waf1 (p21) gene in diverse types of human tumours. *Biochim Biophys Acta* **1263**: 275–280

Williamson MP, Elder PA and Knowles MA (1994) The spectrum of TP53 mutations in bladder carcinoma. *Genes Chrom Cancer* **9**: 108–118

Wright C, Mellon K, Johnston P, Lane DP, Harris AL, Horne CHW and Neal DE (1991) Expression of mutant p53, c-*erb*B-2 and the epidermal growth factor receptor in transitional cell carcinoma of the human urinary bladder. *Br J Cancer* **63**: 967–970

Xu H-J, Cairns P, Hu S-X, Knowles MA and Benedict WF (1993) Loss of RB protein expression in primary bladder cancer correlates with loss of heterozygosity at the RB locus and tumour progression. *Int J Cancer* **53**: 781–784

Yang ZY, Perkins ND, Ohno T, Nabel GN and Nabel GJ (1995) The p21 cyclin-dependent kinase inhibitor suppresses tumorigenicity in vivo. *Nature Med* **1**: 1052–1056

© Cancer Research Campaign 1998